# Associations between higher exposure to potentially morally injurious events and negative posttraumatic cognition trajectories throughout cognitive processing therapy

**DOI:** 10.1002/jts.23179

**Published:** 2025-07-09

**Authors:** Anusha M. Limdi, Daniel R. Szoke, Dale L. Smith, Sarah A. Pridgen, Philip Held

**Affiliations:** ^1^ Department of Psychiatry and Behavioral Science Rush University Medical Center Chicago Illinois USA; ^2^ Department of Psychiatry University of Illinois–Chicago Chicago Illinois USA

## Abstract

Individuals with higher potentially morally injurious event (PMIE) exposure often exhibit elevated levels of negative posttraumatic cognitions (NPCs). Researchers have argued that individuals with moral injury (MI) following PMIE exposure experience more prescriptive NPCs than those without MI. As these prescriptive NPCs may be harder address using cognitive processing therapy (CPT), first‐line posttraumatic stress disorder (PTSD) treatments may not fully address MI. This study evaluated the impact of PMIE exposure on NPC trajectories during intensive CPT for PTSD. We examined NPC trajectories in a group of 738 service members and veterans (SMVs) who participated in a 2‐week CPT‐based intensive PTSD treatment program. Time was a significant predictor of the Posttraumatic Cognitions Inventory (PTCI) score trajectory over treatment, *p* < .001. The interaction between time and PMIE exposure also significantly predicted PTCI trajectories, *p* = .008, such that higher PMIE exposure was related to higher PTCI scores during the first half of treatment; however, by the end of treatment, PTCI scores were visually similar regardless of PMIE exposure. The PTCI subscales (Negative Cognitions About the Self, Negative Cognitions About the World, and Self‐Blame) were also analyzed and resulted in similar associations with time and PMIE exposure as well as with PTCI total score. These findings suggest that intensive CPT appears to be effective in reducing NPCs in SMVs regardless of PMIE exposure. Therefore, even when patients report PMIE exposure, CPT clinicians should continue identifying and targeting NPCs.

Moral injury (MI) is an evolving construct developed to explain the distressing psychosocial, spiritual, religious, and biological reactions to being exposed to potentially morally injurious events (PMIEs; Griffin et al., 2019; Litz et al., 2009). PMIEs can include morally challenging or ethically ambiguous events wherein an individual perpetrates, fails to prevent, or witnesses others act in ways that violate the individual's moral belief system and their expectations of themselves, others, or the world. MI has been observed in several populations, such as service members and veterans (SMVs), health care workers, and first responders (Griffin et al., 2019, 2023; Litz et al., 2009). In military contexts, PMIEs can involve acts of commission (e.g., killing a person, harming civilians, humiliating the enemy) and acts of omission (e.g., failing to prevent the mistreatment of civilians, sexual assault, or harm to others; Frankfurt et al., 2017; Litz et al., 2009). PMIEs may also include betrayal by trusted authority figures, peers, trusted civilians, or institutions in high‐stakes situations (Griffin et al., 2019; Shay, 2014) and non–combat‐related military betrayals, such as military sexual assault (MST; Lopes et al., 2023; Shay, 2014; Stein et al., 2012). In their study of U.S. combat veterans, Wisco et al. (2017) found that 41.8% of veterans endorsed at least one form of PMIE, 25.5% endorsed witnessing transgressions by others, 25.5% endorsed betrayal from trusted authority figures or fellow service members, and 10.8% endorsed perpetrating transgressions.

Exposure to PMIEs may lead to MI, which is hypothesized to include a broad range of symptoms, such as pervasive guilt and shame, feelings of betrayal, depression, spiritual or existential moral outrage or anger, grief, anxiety, self‐condemnation or self‐handicapping, and social problems (i.e., isolation, loss of trust in self or others; Jinkerson, 2016; Wisco et al., 2017). Evidence also suggests that PMIE exposure is associated with posttraumatic stress disorder (PTSD), depressive disorders, and a higher risk of suicide (Litz et al., 2009; Williamson et al., 2018; Wisco et al., 2017). Yet, exposure to PMIEs does not always lead to lasting or persistent MI symptoms (Wisco et al., 2017). Moreover, SMVs face the risk of repeated exposure to PMIEs, which is associated with an increased likelihood of developing persistent MI symptoms (Currier et al., 2021).

Currently, MI is not recognized as a mental disorder in the *Diagnostic and Statistical Manual of Mental Disorders* (5th ed.; text rev.; *DSM*‐5‐*TR*; American Psychiatric Association [APA], 2022) and has been primarily conceptualized and treated as a comorbid presenting problem with PTSD (Griffin et al., 2019). This is due to parallel theoretical conceptualizations of both MI and PTSD, including: (a) exposure to a precipitating traumatic event; (b) stress responses that are automatic, involuntary, and intrusive; and (c) harmful coping mechanisms that lead to persistent and chronic symptoms, impacting overall functioning and well‐being (Currier et al., 2021). However, PTSD diagnoses primarily focus on *DSM‐5‐TR* Criterion A events that are perceived or actual threats to physical safety (APA, 2022; Gonzalez & Martinez, 2014). These traumatic events could qualify as both morally injurious and life‐threatening, causing further challenges in interpreting the associations and distinctions (Stein et al., 2012). Moreover, this diagnostic focus includes PMIEs that meet the criteria but excludes some PMIEs that do not involve life‐threat, serious injury, or sexual violence, such as betrayal by leadership, exposure to human suffering, or traumatic loss (APA, 2022).

Several researchers have attempted to identify whether MI and PTSD have distinct symptom profiles. For example, in a U.S. National Guard sample, Bryan et al. (2018) used exploratory structural equation modeling to document two distinct factors, focusing on MI outcomes and the PTSD Checklist for *DSM‐5* (PCL‐5; Weathers, Litz, et al., 2013). The MI factor was characterized by shame, decreased enjoyment, inward hostility, anger, guilt, and anhedonia symptoms, whereas PTSD was characterized by memory loss, flashbacks, increased startle, nightmares, and insomnia symptoms. Both factors were characterized by depression; however, when accounting for major depressive disorder and PTSD, SMVs who reported MI‐related outcomes had a higher risk of suicidal ideation and attempts as well as anger (Bryan et al., 2018). These findings indicate that MI and PTSD may be similar yet may function as unique constructs.

Furthermore, Litz et al. (2018) examined various trauma types in PTSD treatment‐seeking SMVs and found that SMVs who endorsed perpetration‐based MI as their traumatic event had higher levels of guilt‐ and self‐blame–related cognitions and intrusion symptoms relative to those who reported life‐threat traumatic events. In addition, SMVs who witnessed others violate their moral standards endorsed more peri‐ and posttraumatic betrayal emotions relative to those who experienced life threat trauma or a serious injury. Evidence suggests that negative posttraumatic cognitions (NPCs) regarding responsibility and the context of the event can mediate the association between PMIE exposure and MI. In one study, SMVs who endorsed perpetration‐based MI transgressions and felt they were directly responsible reported guilt and shame, beliefs that they were unlovable, feelings of being unforgivable, feeling incapable of making moral decisions, and self‐handicapping behaviors (Currier et al., 2017). In contrast, SMVs who endorsed betrayal or witnessed others’ moral transgressions and did *not* feel directly responsible experienced anger, moral disgust, a lack of trust, and revenge fantasies. Taken together, the sequelae of trauma‐related negative cognitions following MI may be distinct to those typical of PTSD and require further exploration of whether current first‐line PTSD therapies can be effective for both.

Cognitive and emotional processing theories describe that negative, stable, internal, and global appraisal following traumatic events may function as a mechanism in the development and maintenance of PTSD and MI (Ehlers & Clark, 2000; Held et al., 2017). Investigators postulate that MI develops due to NPCs related to the PMIEs and maladaptive meaning making following exposure (Held et al., 2017). Further evidence also suggests that NPCs may mediate the association between PMIE exposure and PTSD severity. As such, evidence‐based psychotherapies, such as cognitive processing therapy (CPT; Resick et al., 2024), that target NPCs (e.g., self‐blame, negative cognitions about the self and world) through Socratic questioning and other cognitive restructuring techniques can be effective in treating PTSD in veterans with MI (Held et al., 2018). Furthermore, Held et al. (2021) demonstrated that PMIE exposure and index trauma type (i.e., PMIE vs. non‐PMIE) did not impact outcomes of a 3‐week intensive treatment program (ITP) for PTSD in a sample of veterans.

Similar outcomes, regardless of PMIE exposure, may be due to the use of Socratic dialogue in CPT, which targets the falsifiability of “distorted” or “exaggerated” NPCs and is particularly effective with descriptive cognitions (Resick et al., 2024). Descriptive cognitions are beliefs that make claims about the nature of oneself, others, or the world as well as causal relationships (e.g., “The world is never a safe place,” “I am a monster for what I've done,” “The military does not care about women service members”; Ehlers et al., 2005; Farnsworth et al., 2017). Through Socratic dialogue, these beliefs can be evaluated and corrected through cognitive restructuring. However, Farnsworth (2019) argued that individuals with MI often endorse violations of prescriptive cognitions, which may be more difficult to falsify through the Socratic process. Prescriptive cognitions (e.g., “War should never happen,” “Women and children should always be protected from war,” “The world should be a safe place”) concern what the individual believes morally ought to be and are unique to an individual's moral standards and preferences for themselves, others, and the world. It has been argued that these cognitions are considered “inherently unfalsifiable” and moral in nature and may not be accurately evaluated as exaggerated or distorted; therefore, these beliefs and related symptoms may not respond to CPT (APA, 2022; Farnsworth, 2019).

Some researchers also argue that CPT may be insufficient for treating accurate self‐blame NPCs following MI (Gray et al., 2017; Harris et al., 2024; Maguen et al., 2017). Gray et al. (2017) expressed concern regarding CPT's ability to address self‐blame cognitions in situations in which an SMV has intentionally transgressed their moral codes (e.g., killing a noncombatant, humiliating the enemy). The authors argue that for these events, self‐blame can be accurate and appropriate and may not be falsifiable or exaggerated; therefore, it may remain improperly addressed through Socratic dialogue. Furthermore, Litz et al. (2021) developed a therapeutic intervention, adaptive disclosure, to explicitly address unique etiological factors and outcomes related to combat trauma and war zone events and found it to be no less effective than established first‐line PTSD therapy for deployed U.S. Marines and sailors. The authors measured NPCs using the Posttraumatic Cognitions Inventory (PTCI; Foa et al., 1999), which encompasses a predetermined list of trauma‐related beliefs commonly held by trauma survivors. Currently, there are no measures to assess cognitions associated specifically with PMIEs. To advance this ongoing discussion in the literature, it is essential to examine the effectiveness of CPT for PTSD on reducing NPCs in individuals with higher degrees of PMIE endorsement.

Exploring the association between PMIE exposure on NPCs throughout PTSD treatment may reveal distinct trajectories of NPC change for SMVs with varying levels of exposure to PMIEs. For example, SMVs with higher degrees of PMIE exposure may report less self‐blame reductions, whereas SMVs with lower PMIE exposure levels may experience larger improvements in self‐blame during treatment. Understanding these trajectories is critical for determining whether first‐line EBTs are effective in reducing harmful NPCs in SMVs who report higher levels of exposure and distress related to MI and PTSD.

Given the existing gap in the literature and concerns raised by MI researchers (Gray et al., 2017; Litz et al., 2009) regarding the limitations of CPT in addressing maladaptive beliefs among individuals experiencing MI, we examined the associations between PMIE exposure and NPC trajectories among SMVs during a CPT‐based 2‐week ITP for PTSD, assessing NPCs using the PTCI total score and PTCI Self‐Blame, Negative Cognitions About the Self (PTCI‐Self), and Negative Cognitions About the World (PTCI‐World) subscales. The first aim of the study was to identify associations between these three types of NPCs (i.e., self‐blame, self‐related negative cognitions, world‐related negative cognitions) and PTSD in SMVs with varying levels of PMIE exposure. We hypothesized that higher endorsement of PMIE exposure and related distress would be positively associated with higher baseline NPC severity. The second aim was to evaluate the associations between trajectories of NPC severity change and PMIE exposure severity. We predicted that higher levels of PMIE endorsement would be associated with less change in all three types of NPCs during the ITP.

## METHOD

### Participants

This study sample consisted of 738 SMVs who participated in a 2‐week CPT‐based ITP for PTSD at the Road Home Program: Center for Veterans and Their Families (RHP) at Rush University Medical Center in Chicago (Illinois, United States). Approximately 54% of participants self‐identified as male (*n* = 398), and 46.1% (*n* = 340) identified as female. The mean participant age was 44.7 years (*SD* = 10.2; range: 20–81). The majority of the sample identified as White (64.6%) and non‐Hispanic or Latino (82.7%), were honorably discharged (86.7%), and served after September 11, 2001 (9/11; 83.5%). The most commonly reported branch of service was the U.S. Army (43.0%). Additional sample characteristics are displayed in Table 1.

**TABLE 1:** Demographic and clinical characteristics of the sample

Variable	*n*	%	*M*	*SD*
Age (years)	738		44.74	10.20
Sex				
Female	340	46.10		
Male	398	53.19		
Ethnicity				
Non‐Hispanic	620	82.70		
Hispanic	128	17.30		
Race				
American Indian or Alaska Native	6	0.80		
Asian	22	3.00		
Black or African American	162	22.00		
Native Hawaiian or Pacific Islander	9	1.20		
White	477	64.60		
Other	62	8.4		
Service era				
Before September 11, 2001	122	16.53		
After September 11, 2001	616	83.35		
Cohort				
Combat	395	53.50		
MST	343	46.48		
PTCI total, baseline	738		149.60	36.15
PTCI total, posttreatment	644		121.90	41.77
PTCI‐Self, baseline	738		4.40	1.21
PTCI‐Self, posttreatment	644		3.50	1.44
PTCI Self‐Blame, baseline	738		3.64	1.68
PTCI Self‐Blame, posttreatment	644		3.12	1.54
PTCI World, baseline	738		5.55	1.10
PTCI World, posttreatment	644		4.68	1.33
MIES total, baseline	738		36.54	11.14

*Note*: *N* = 738. PTCI = Posttraumatic Cognitions Inventory; PTCI‐Self = PTCI Negative Cognitions About the Self subscale; PTCI‐World = PTCI Negative Cognitions About the World subscale; MST = military sexual trauma;, MIES = Moral Injury Events Scale.

### Procedure

The data for the present study were collected from July 2020 to March 2024 at the Road Home Program, which provides psychological care to SMVs regardless of discharge status, branch of service, or length of service. SMVs participate in a 2‐week in‐person ITP in either the combat trauma or MST cohort, depending on their self‐identified index traumatic event. The 2‐week ITP includes two 50‐min individual CPT sessions per day, with a total of 16 individual therapy CPT sessions. Participants also receive six group therapy sessions, four group mindfulness‐based sessions, and several adjunctive services (e.g., acupuncture, art therapy, case management), all free of cost, including transportation, housing, and meals. For additional information about the ITP, please refer to (Held et al., 2023). Both cohorts are mixed‐gender, and the MST track offers an additional service of pelvic wellness. All study procedures were approved by the Rush University Medical Center Institutional Review Board.

All treatment‐seeking SMVs completed an intake process with semistructured interviews and diagnostic and self‐report assessments before acceptance into the ITP. SMVs must meet the diagnostic criteria for a primary diagnosis of PTSD, confirmed using the Clinician‐Administered PTSD Scale for *DSM‐5* (Weathers, Blake, et al., 2013). SMVs who endorsed active suicidality or homicidality with clear intent and a plan, severe nonsuicidal self‐harm, or unmanaged mania or psychosis in the last 3 months were excluded from the program. Additionally, SMVs with substance use that could not be discontinued without medical attention or the risk of withdrawal were ineligible for the ITP. Both the combat and MST cohorts were included in the sample.

### Measures

#### Demographic characteristics

During the intake evaluation, age, sex, post‐9/11 military service, ethnicity, race, marital status, service status, discharge status, and branch of service were collected.

#### PMIE exposure

The Moral Injury Events Scale (MIES; Nash et al., 2013) is a nine‐item measure that assesses exposure to PMIEs and related distress. Items are rated on a 6‐point Likert scale ranging from 1 (*strongly disagree*) to 6 (*strongly agree*); higher scores indicate higher levels of PMIE exposure and MI‐related distress. The MIES has demonstrated excellent internal reliability in ground combat Marines (Cronbach's α = .90; Nash et al., 2013) and favorable internal validity and fair internal consistency reliability in SMV samples (Cronbach's α = .79; Bryan et al., 2016). There is no suggested clinical cutoff score for the MIES. In this current study, the MIES was administered only at baseline to measure PMIE exposure at any time since joining the military. In the current sample, the MIES demonstrated very good internal reliability, Cronbach's α = .85.

#### Trauma‐related cognitions

The PTCI (Foa et al., 1999) is a 33‐item measure of self‐reported negative trauma‐related cognitions that develop following exposure to a traumatic event. The PTCI includes 21 items assessing negative cognitions about oneself (PTCI‐Self; e.g., “I have permanently changed for the worse”), seven items assessing negative cognitions about the world (PTCI‐World; e.g., “The world is a dangerous place”), and five items assessing self‐blame (PTCI‐Self‐Blame; e.g., “The event happened because of the way I acted”). Items are rated on a 7‐point Likert scale ranging from 1 (*totally disagree*) to 7 (*totally agree*); higher scores indicate higher levels of NPC severity. The PTCI has demonstrated excellent internal consistency and reliability and has been used in SMV populations with PTSD to assess NPCs (Held et al., 2017; Raab et al., 2015; Splaine et al., 2023). The PTCI was administered at baseline (pretreatment), Day 1, Day 4, and posttreatment (Day 9) to measure daily NPC severity. In the current sample, the PTCI demonstrated excellent internal reliability across assessments, Cronbach's αs = .95–.97.

#### PTSD symptoms

The PCL‐5 (Weathers, Litz, et al., 2013) is a 20‐item self‐report measure that assesses PTSD symptom severity according to the *DSM‐5* criteria, including intrusions, avoidance, maladaptive NPCs, and arousal. Symptoms are rated on a 5‐point scale ranging from 0 (*not at all*) to 4 (*extremely*), with a possible score range of 0–80 and higher scores indicating higher levels of symptom severity. The PCL‐5 has been found to be psychometrically sound in SMV populations (Bovin et al., 2016). The established clinical cutoff for probable PTSD is a score of 31–33 (Bovin et al., 2016; Weathers, Litz, et al., 2013). In the current study, the PCL‐5 was administered at baseline (pretreatment) to measure past‐month symptom severity and at posttreatment (Day 9) to assess past‐week symptom severity. SMVs were asked to report PTSD symptoms related to the index traumatic event chosen to be the focus of CPT. In the current sample, the PCL‐5 demonstrated excellent internal consistency, Cronbach's αs = .92–.96.

#### Depressive symptoms

The nine‐item Patient Health Questionnaire (PHQ‐9; Kroenke et al., 2001) is a self‐report measure that was used to assess depression symptom severity over the last 2 weeks. Items are rated on a scale of 0 (*not at all*) to 3 (*nearly every day*) and summed to create a total depressive symptom score. Higher PHQ‐9 total scores indicate higher levels of depressive symptom severity, with a cutoff score of 10 or higher indicating clinically significant symptoms (i.e., a probable depressive disorder). The PHQ‐9 is considered a reliable and valid measure of depressive symptom severity (Kroenke et al., 2001). In the current study, the PHQ‐9 was administered at baseline (pretreatment) and posttreatment (Day 9) to measure severity of symptoms over the past 2 weeks. In this sample, the PHQ‐9 demonstrated good internal reliability, Cronbach's α = .84–.87.

### Data analysis

We used descriptive statistics to characterize our sample by calculating means, ranges, and standard deviations for our demographic, independent, and dependent variables. Furthermore, we utilized linear mixed‐effects regression models (LMMs) to examine PMIEs as a predictor of NPC change over time in the 2‐week ITP for SMVs with PTSD. Linear mixed‐effects regression models are particularly appropriate for longitudinal analyses due to their ability to handle some missing data and their flexibility in variance and covariance structure. In addition to total PTCI scores, we examined predictions of PTCI subscale scores (i.e., PTCI‐Self‐Blame, PTCI‐Self, PTCI‐World) in separate models. To address family‐wise error in the regression models predicting these four outcomes, we utilized a Bonferroni correction. All models were adjusted for sex, race, ethnicity, age, post‐9/11 status, cohort type, and PCL‐5 and PHQ‐9 scores at baseline. In addition to the main effects of MIES scores, interactions between MIES scores and time were examined to determine whether PTCI total score and subscale score trends differed over time based on PMIE exposure levels. Analyses were conducted in Stata (Version 17). Statistical significance was determined using an alpha level of .05.

## RESULTS

Descriptive statistics were calculated at baseline for the MIES and at baseline and posttreatment for the PTCI. At baseline, the mean MIES score was 36.54 (*SD* = 11.14; range: 9–54), and the mean PTCI score was 149.6 (*SD* = 36.15, range: 40–231). After treatment completion, participants showed a mean PTCI score of 121.9 (*SD* = 41.77; range: 33–226). Paired *t* tests reveal a statistically significant reduction in total PTCI scores from baseline to posttreatment, Δ*M* = 28.78, *p* < .001, *d* = 0.73.

**FIGURE 1:**
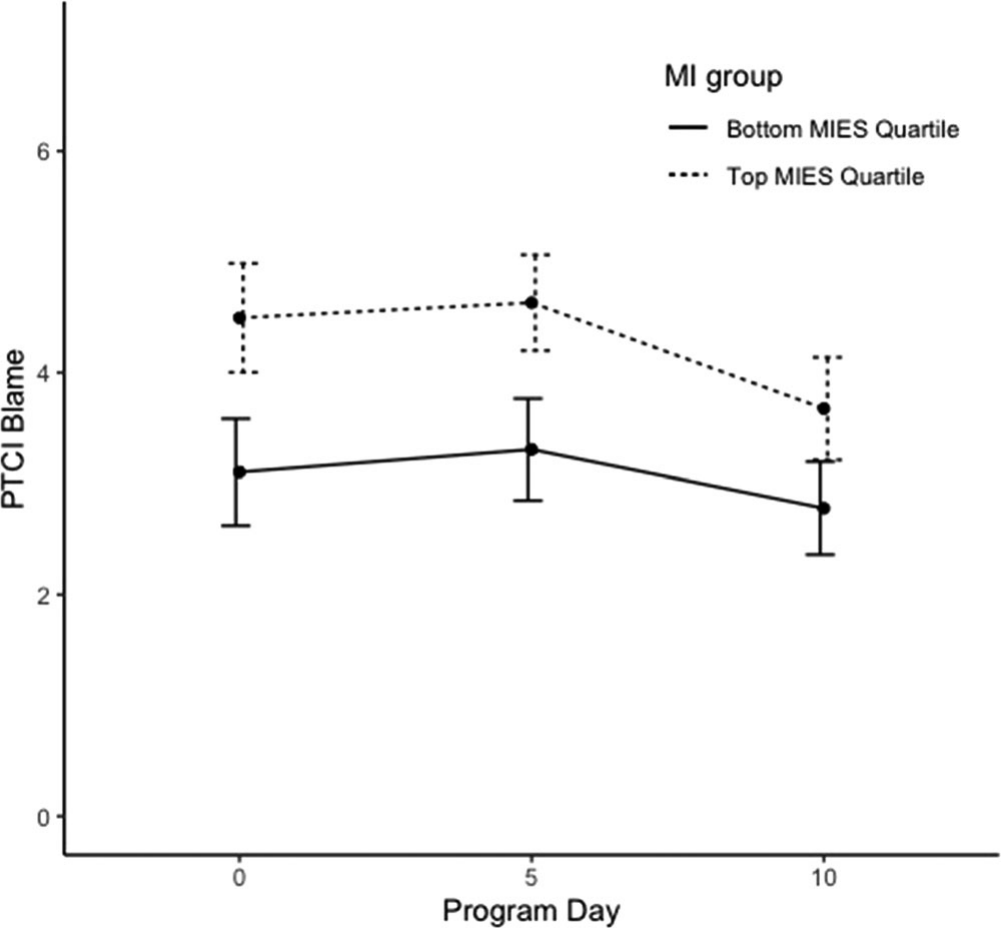
*Blame‐related negative posttraumatic cognition trajectories among service members and veterans in a 2‐week intensive treatment program for posttraumatic stress disorde* *r* *Note*: Blame‐related negative posttraumatic cognitions were assessed using the Posttraumatic Cognitions Inventory (PTCI) Self‐Blame subscale. MIES = Moral Injury Events Scale.

**FIGURE 2:**
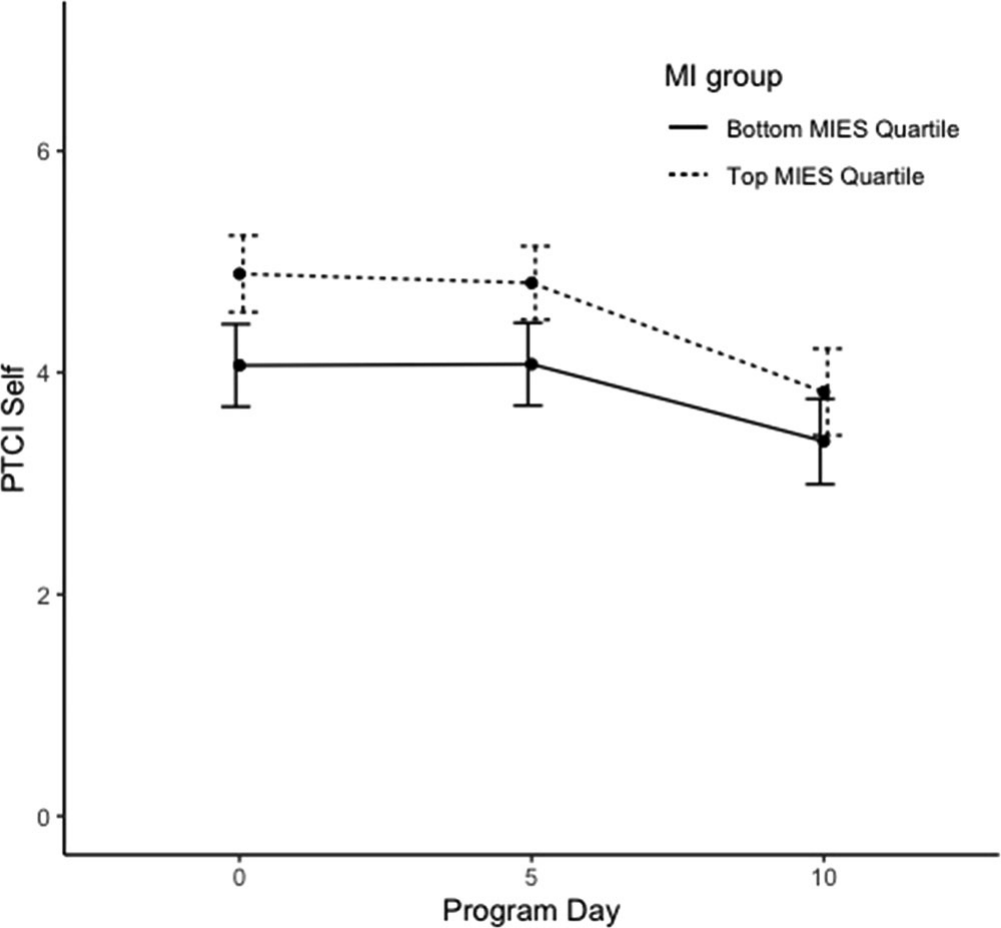
Self‐related negative posttraumatic cognition trajectories among service members and veterans in a 2‐week intensive treatment program for posttraumatic stress disorder *Note*: Self‐related negative posttraumatic cognitions were assessed using the Posttraumatic Cognitions Inventory (PTCI) Negative Cognitions About the Self subscale. MIES = Moral Injury Events Scale.

**FIGURE 3:**
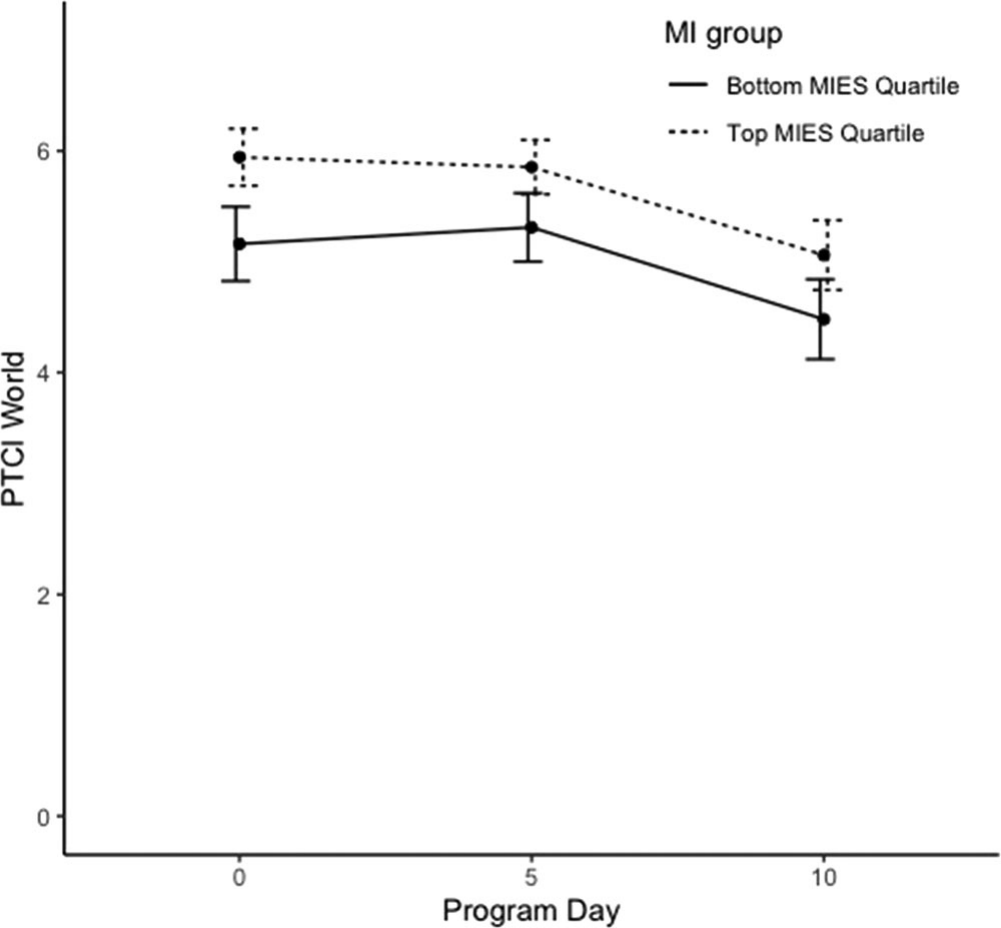
World‐related negative posttraumatic cognition trajectories among service members and veterans in a 2‐week intensive treatment program for posttraumatic stress disorder *Note*: World‐related negative posttraumatic cognitions were assessed using the Posttraumatic Cognitions Inventory (PTCI) Negative Cognitions About the World subscale. MIES = Moral Injury Events Scale.

Linear mixed‐effects regression models predicting PTCI scores indicated that both linear and quadratic time trends were significant, *p*s < .001, though no demographic covariates were significant predictors of PTCI scores over time (see Table 2). The time‐varying covariates of PTSD symptom severity and depressive symptom severity were both significant predictors of PTCI score, indicating that improvements in these conditions were associated with improvements in NPC severity, *p*s < .001. A higher baseline total MIES score was a significant predictor of PTCI scores over time, indicating that higher levels of PMIE exposure were associated with higher total PTCI scores during the program. Additionally, the interaction between the MIES and time was also significant, *p* = .008, suggesting that trends across time differed based on these values. Although participants with higher MIES scores generally began the program with higher levels of NPC severity, they appeared to improve slightly more during the program to the extent that end‐of‐program NPCs were similar regardless of PMIE exposure at baseline (see Figures 1, 2, 3). The same trends existed when predicting both the PTCI‐Self and PTCI‐Self‐Blame subscales, though only the main effect of PMIE exposure, and not its interaction with time, was significant for the PTCI‐World subscale (see Table 3).

**TABLE 2:** Predictors of Posttraumatic Cognitions Inventory (PTCI) trajectory among participants

Variable	*B*	95% CI	*p*
Time	4.70	[3.52, 5.89]	< .001^a^
Time	−0.62	[−0.69, −0.55]	< .001^a^
Sexª	1.75	[−3.79, 7.29]	.537
Race	−3.75	[−7.42, −0.08]	.045
Ethnicity	1.91	[−2.65, 6.47]	.412
Age	−0.19	[−0.38, 0.01]	.059
Service era	−4.13	[−9.47, 1.21]	.130
Cohort type	2.96	[−2.65, 8.56]	.301
PCL‐5	0.93	[0.74, 1.11]	< .001^a^
PHQ‐9	1.69	[1.24, 2.14]	< .001^a^
MIES total	0.52	[0.35, 0.69]	< .001^a^
MIES Total x Time	−0.04	[−0.06, −0.01]	.008^a^

*Note*: Reference categories were male; White, non‐Hispanic; military service before September 11, 2001; and military sexual trauma cohort. MIES = Moral Injury Events Scale; PCL‐5 = PTSD Checklist for *DSM‐5*; PHQ‐9 = Patient Health Questionnaire–9.

^a^ Significant following Bonferroni correction for familywise error.

**TABLE 3:** Models Predicting Posttraumatic Cognitions Inventory (PTCI) subscale scores

	PTCI‐Self			PTCI‐World			PTCI‐Self‐Blame		
Variable^a^	*B*	95% CI	*p*	*B*	95% CI	*p*	*B*	95% CI	*p*
MIES total	0.10	[0.01, 0.02]	< .001^b^	0.009	[.003, .014]	.001^b^	0.03	[0.02, 0.03]	< .001^b^
MIES Total x Time	−0.001	[−0.002, −0.0002]	.012^b^	−0.0006	[−0.001, 0.0003]	.189	−0.002	[−0.003, −0.0008]	.001^b^

*Note*. PTCI = Posttraumatic Cognitions Inventory; PTCI‐Self = PTCI Negative Cognitions About the Self subscale; PTCI‐World = PTCI Negative Cognitions About the World subscale; MIES = Moral Injury Events Scale.

^a^ Significant following correction for multiple comparisons.

^b^ Models were adjusted for demographic variables and baseline PTSD Checklist for *DSM‐5* Patient Health Questionnaire–9 scores.

## DISCUSSION

This study examined the degree to which PMIE exposure impacted NPCs during a CPT‐based ITP. Consistent with our hypothesis, we found that SMVs with higher PMIE exposure, as measured using the MIES, began treatment with significantly higher baseline levels of NPC severity, as measured using the PTCI. This pattern aligns with existing literature indicating that exposure to PMIEs and engagement in moral transgressions contribute to increased self‐blame and negative evaluations of oneself and the world (Currier et al., 2017; Held et al., 2018, 2017; Litz et al., 2018).

Furthermore, we examined the NPC trajectories during the program. There was an overall negative association between NPCs and time; however, the strength of the association varied over the course of the program. We also explored the association between NPC trajectories and PMIE exposure. The initial negative impact of PMIE exposure on NPC overall severity significantly reduced over time; therefore, SMVs reported experiencing improvement in maladaptive cognitions as the treatment progressed. This interaction effect was present for both the PTCI‐Self‐Blame and PTCI‐Self subscales, as well as PTCI total scores. Therefore, PMIE exposure demonstrated a stronger association with these subscales and total PTCI scores at the start of treatment, which weakened by the end of treatment.

In contrast, the interaction between time and negative cognitions about the world (i.e., PTCI‐World) was not significant, indicating that the association between higher PMIE exposure and negative world‐related beliefs did not vary over time; higher PMIE exposure continued to predict higher negative world‐related beliefs in SMVs throughout treatment. This indicates that the strong initial negative impact of PMIE exposure on world‐related beliefs remained throughout treatment. Therefore, it is plausible that the existing intensive CPT timeframe might not be sufficient to fully address these negative world‐related beliefs in SMVs with higher degrees of PMIE exposure. A standard or longer course of treatment may have allowed for more improvement in negative cognitions about the world. Additionally, in SMVs who have experienced higher levels of PMIE exposure, negative cognitions about the world may be more prescriptive in nature and, resultingly, may have been more difficult to fully address using the Socratic method over the course of the program. However, it is important to note that negative world‐related beliefs greatly reduced throughout treatment and remained only slightly elevated at posttreatment for SMVs with higher PMIE exposure levels compared to those with lower levels of PMIE exposure. The substantial reduction in negative world‐related beliefs by the end of treatment demonstrates that CPT is effective for SMVs with PTSD.

Additionally, our analysis revealed that the MIES was associated with the PTCI even when controlling for PTSD and depressive symptom severity, suggesting that higher PMIE exposure contributes uniquely to NPC levels throughout treatment. Consistent with previous findings (Szoke et al., 2024), demographic covariates did not predict changes in PTCI scores over time, indicating that age, sex, race, ethnicity, service era, and cohort type (combat trauma or MST) had no significant effect on PTCI score change trajectories throughout the program.

Our findings appear to challenge the discourse that CPT for PTSD is insufficient for addressing MI‐related outcomes (Farnsworth, 2019; Gray et al., 2017; Harris et al., 2024; Maguen et al., 2017), as commonly held NPCs in individuals with higher PMIE exposure, as measured using the PTCI, can significantly improve in response to CPT for PTSD. This underscores CPT's versatility and reinforces its value as a comprehensive treatment for PTSD, inclusive of individuals with MI‐related experiences. Based on these findings, clinicians who identify an SMV with PTSD and MI‐related concerns are encouraged to apply CPT for PTSD as a first‐line treatment, as those with higher versus lower PMIE exposure do not ultimately differ in NPC change during CPT.

Furthermore, clinicians in the current study were expected to adhere to the manual guidelines for CPT for PTSD and did not explicitly attempt to challenge prescriptive cognitions that individuals with MI felt they had uniquely violated (e.g., women and children should be protected in war; Farnsworth, 2019; Resick et al., 2024). Instead, clinicians likely focused on addressing related underlying assimilated beliefs (e.g., “I should have done more to protect the women and children from war”) and used Socratic questioning to replace them with adaptive alternative beliefs (e.g., “I did the best I could with the resources I had to protect women and children”). The results indicate that this method may have been successful in ameliorating the elevated NPCs that participants with higher PMIE exposure reported at the outset of treatment.

Additionally, we encourage CPT clinicians to continue assessing MI and NPCs in SMVs undergoing CPT for PTSD. In our study, higher PMIE exposure was associated with higher initial levels of NPCs; however, it did not continue to predict higher levels of NPCs by the end of treatment, especially regarding negative cognitions about oneself and self‐blame. Therefore, we recommend that CPT clinicians continue to address these NPCs as outlined in the CPT manual (Resick et al., 2024). However, negative world‐related cognitions in SMVs with higher PMIE exposure may demonstrate less change during CPT treatment. Clinicians should monitor and discuss world‐related NPCs with SMVs throughout treatment to support cognition change, as evidence suggests that formally monitoring patient progress significantly benefits individuals with a poor initial response to treatment, and continuous monitoring can provide clues regarding the remaining NPCs that need to be challenged before ending treatment (Lambert et al., 2003). This approach may help address world‐related NPCs more effectively by the end of treatment.

It is important to acknowledge the limitations of this study. First, to be eligible for our program, SMVs require a PTSD diagnosis related to combat trauma or MST. As previously mentioned, PMIEs do not always meet the criteria for *DSM‐*5 Criterion A events; consequently, we cannot assess the effectiveness of CPT for psychopathology related to MI that does not manifest as PTSD. Second, all SMVs participated in a 2‐week CPT program that included several adjunctive services, such as mindfulness meditation, art therapy, acupuncture, and psychoeducational groups; therefore, it is possible that these adjunctive services contributed to the overall change seen in NPCs in addition to CPT. Third, SMVs self‐reported NPCs from a predetermined list of common trauma‐related cognitions that are specific to the *DSM‐5* diagnostic criteria for PTSD rather than NPCs that are specific to the unique index traumatic event and the individual receiving treatment. Therefore, it is possible that NPC change was not accurately captured beyond the items contained on the PTCI. Fourth, it is important to recognize that a higher MIES score does not directly translate to MI, as the MIES captures both exposure to transgressive events and the psychological distress related to the event; therefore, the conclusions of this study may be applicable only to self‐reported MIES scores and not the wider concept of MI. Lastly, causal conclusions cannot be made regarding the association between higher levels of PMIE exposure and NPC trajectories during the program.

Future studies should explore how negative world‐related beliefs develop and persist in individuals with higher levels of PMIE exposure. In addition, researchers could investigate the mechanisms through which PMIEs contribute to negative world‐related beliefs and identify factors that make these beliefs more resistant to change in CPT. Additionally, conducting longitudinal studies could provide deeper insights into the long‐term effects of CPT on the association between PMIE exposure and NPC change at longer‐term follow‐up time points. Furthermore, assessing whether improvements in negative cognitions about oneself and self‐blame are sustained over time and whether negative beliefs about the world eventually reduce would provide further evidence of whether additional interventions may be needed for SMVs with PTSD and higher PMIE exposure. Previous research indicates that depressive and PTSD symptom severity are greatly reduced following 2‐week and 3‐week intensive CPT for PTSD (Held et al., 2024); however, further examinations of whether world‐related NPCs are still impacting SMVs in their daily life following treatment are warranted. Furthermore, research can explore whether individuals with higher PMIE exposure can retain some negative beliefs about the world and still experience improvement in PTSD and MI‐related distress. Future research could provide further evidence of whether the difference in rates of negative beliefs about the world is clinically meaningful and requires additional intervention for individuals with higher PMIE exposure levels.

Furthermore, CPT effectively targets harmful cognitions that cause distress in individuals who have experienced various types of traumatic events. This intervention may also be beneficial for people who have endured non–Criterion A PMIEs, as these populations also endorse higher NPCs. Future research analyzing a sample of individuals with MI stemming from a non–Criterion A traumatic event, regardless of PTSD diagnostic status, who are undergoing CPT could provide valuable insights into CPT outcomes specific to PMIEs. This approach would help characterize the effectiveness of CPT in addressing NPCs associated with morally challenging traumatic events. Furthermore, future research should work to develop a measure like the PTCI that specifically captures MI‐related cognitions (e.g., prescriptive beliefs). This measure would help further characterize the impact of CPT on MI‐related cognitions trajectories.

In summary, our study contributes to the growing body of evidence supporting the effectiveness of intensive CPT for reducing NPCs in SMVs with varying degrees of PMIE exposure. Our research challenges previous assumptions that CPT may be insufficient for addressing NPCs in individuals with PTSD and higher levels of PMIE exposure. The current study demonstrates that significant cognitive improvements can be achieved within the existing CPT framework, even for individuals who endorse higher PMIE exposure. Moreover, clinicians should continue to address NPCs according to the CPT manual for PTSD, as this intervention results in beneficial improvements for SMVs with varying degrees of PMIE endorsement (Resick et al., 2024). However, negative world‐related cognitions may demonstrate less change during treatment, and clinicians should monitor and discuss world‐related NPCs with SMVs who endorse higher levels of PMIE exposure throughout treatment as needed to support cognition change in those with poor initial response to treatment.

## AUTHOR NOTE

Philip Held receives grant support from the Department of Defense (W81XWH‐22‐1‐0739, TP230421, TP230385), Wounded Warrior Project, United Services Automobile Association (USAA)/Face the Fight, and the Crown Family Foundation.

The content is solely the responsibility of the authors and does not necessarily represent the official views of the Department of Defense, Wounded Warrior Project, USAA, or any other funding agency. All authors declare that they have no competing interests.

We would like to thank the participating veterans and their families, as well as Wounded Warrior Project, for their support of the Road Home Program and Warrior Care Network. We also wish to acknowledge the Road Home Program administrators, research assistants, and clinicians for their contributions to this work.

## OPEN PRACTICES STATEMENT

The study reported in this article was not formally preregistered. Neither the data nor the materials have been made available on a permanent third‐party archive; requests for the data or materials can be sent via email to the senior author at philip_held@rush.edu.
